# When transgenerational trauma meets family secrets : a case study in the Tunisian population

**DOI:** 10.1192/j.eurpsy.2025.1282

**Published:** 2025-08-26

**Authors:** I. Ben Salem Planchaud, M. Ben Hmida

**Affiliations:** 1 Centre municipal de santé, Saint Fargeau Ponthierry; 2 Psychology, Université d’Angers, Angers, France

## Abstract

**Introduction:**

More than any other field in medicine, psychiatry is marked by an individual’s life experiences and the cultural context surrounding them. When a patient presents a set of somatic and seemingly unrelated symptoms that can’t be explained by an underlying physical condition, the diagnosis can become challenging. However, when these symptoms are seen through the lense of their particular cultural context and their traumatic family history, these manifestations start to make sense.

**Objectives:**

This paper aims to examine the relationship between transgenerational trauma and somatic manifestations without an underlying physical condition in an adult female partient N.

**Methods:**

In order to investigate the psychological functioning of the patient N, we conducted a semi-structured interview followed by a set of psychological tests. We administered the Draw-a-Family Picture Test, the Rorschach Test and the Thematic Apperception Test (TAT). We also analysed the patient’s diary entries.

**Results:**

N initially came to us with unexplained somatic symptoms and a generally depressive mood, finding it difficult to discuss certain topics like her sexual issues. The various elements we have collected through the tests and the interviews with the patient N have shed light on an event that occured within the father’s family, an incestuous rape. This event was the cause of the family’s breakdown, and we believe that the depressive symptoms, suicide attempts, as well as the incestuous atmosphere related to the patient N and her family are connected to this event and have continued to be unconsciously transmitted across generations.

**Conclusions:**

This case study showed the importance of taking family history and cultural context into consideration when examining psychological functioning. Family secrets and seemingly unrelated past events can cause a transgenerational trauma affecting the descendants mental and physical health.

**Disclosure of Interest:**

None Declared

